# Supplementary material for: Simulating multiple faceted variability in single cell RNA sequencing
Source: Nat Commun. 2019 Jun 13;10:2611. doi: 10.1038/s41467-019-10500-w (PMC6565723; doi:10.1038/s41467-019-10500-w)
Supplement: Supplementary file 1 — Supplementary Information [file 41467_2019_10500_MOESM1_ESM.pdf]

Supplementary Information

Simulating multiple faceted variability in single cell RNA sequencing

Zhang *et al.*

Supplementary Figures

Supplementary Figure 1

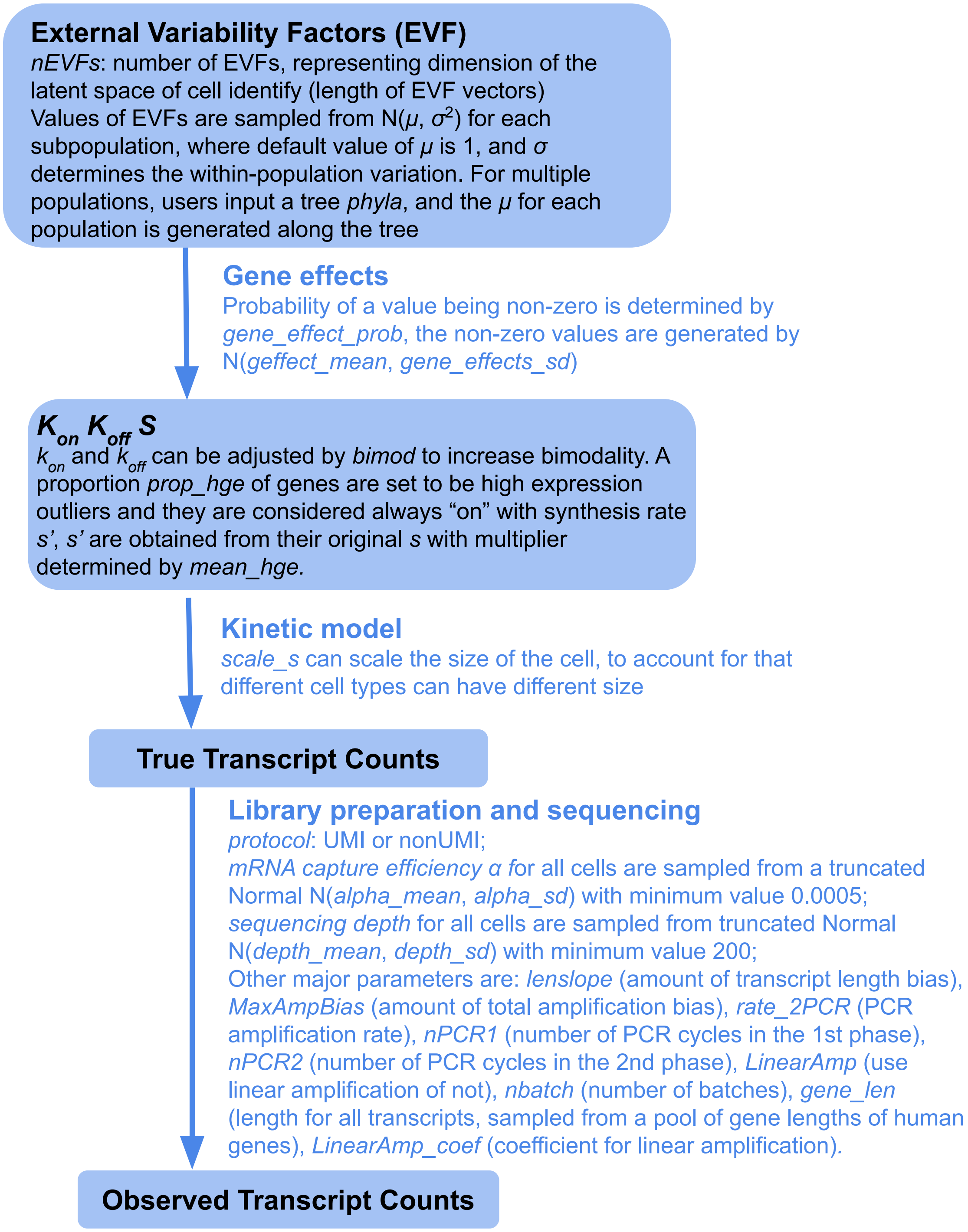

Supplementary Figure 1 A flow chart of the process of SymSim generating the observed counts, with parameters involved in each step described.

Supplementary Figure 2

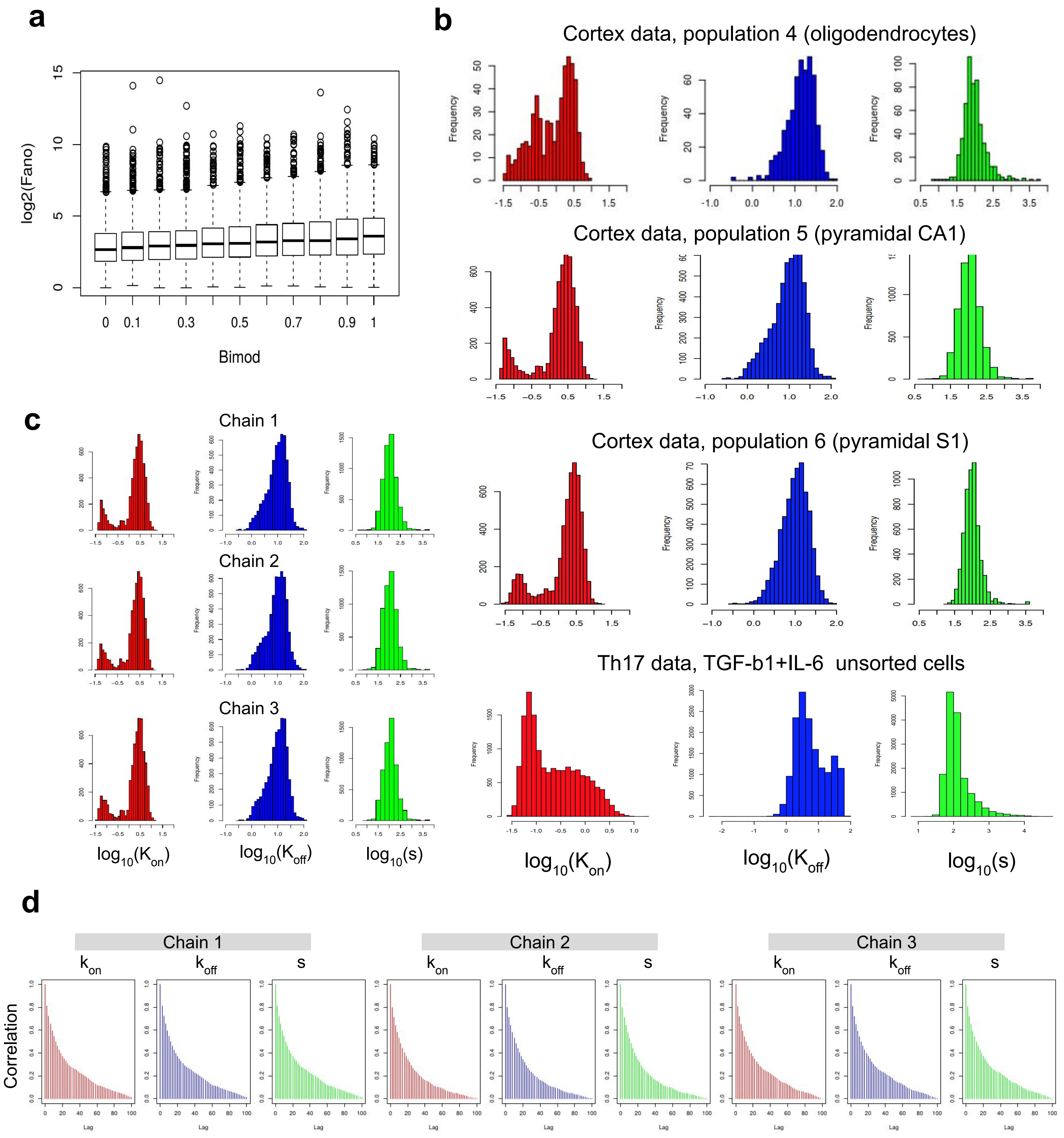

Supplementary Figure 2 The Bimod parameter affects the Fano factor of genes. Results and diagnose plots for kinetic parameter estimation. (**a**) The Fano factor of genes across cells with different values for *Bimod*. (**b**) Distribution of kinetic parameters estimated from different experimental data. (**c**) Distribution of kinetic parameters estimated for the same data (UMI cortex dataset, population pyramidal CA1, imputed with scVI) using different starting values to obtain multiple MCMC chains. (**d**) Auto-correlation diagnose plots for the three chains in (c). The correlation values are average over all genes. For each lag value *k*, correlation is calculated between x_t and x_{t+k}, where x_t is a sample from MCMC at step *t*.

Supplementary Figure 3

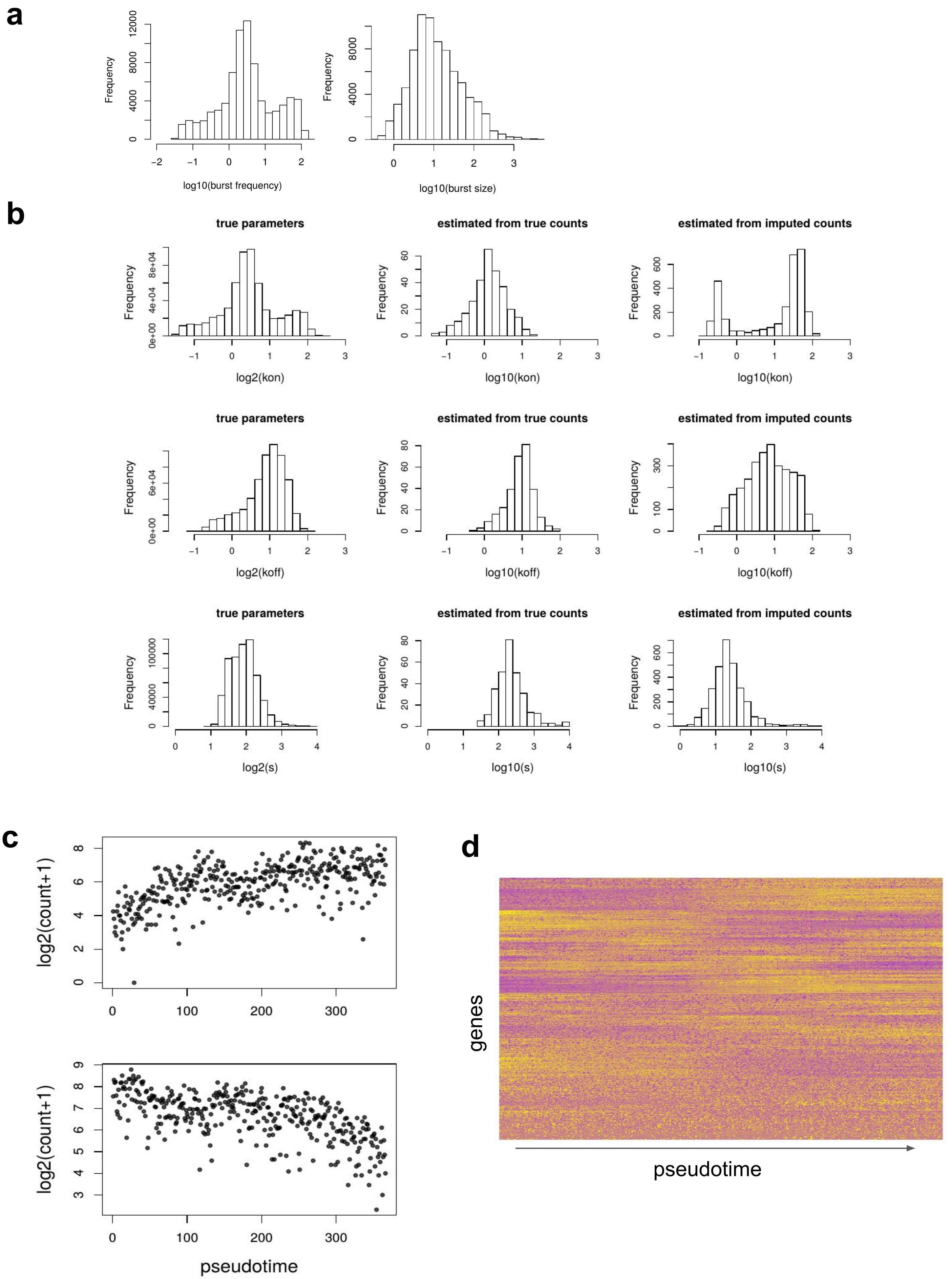

Supplementary Figure 3 Comparisons of results from kinetic parameter estimation procedure. Gene-expression change over pseudotime in continuous populations. (**a**) distributions of kinetic parameters shown in Figure 2b in forms of burst frequency (k_{on}) and burst size (s/k_{off}). (**b**) distributions of burst frequency (k_{on}) and burst size (s/k_{off}) reported by Larsson *et al* [46] in mouse fibroblasts. (**c**) Comparison of distributions of true kinetic parameters (left column), parameters estimated from true counts (middle column), and parameters estimated from imputed counts (right column). True counts and observed counts of five populations are obtained through running SymSim with the tree shown in Figure 3a. (**d**) The gene expression of two DE genes along the pseudotime in continuous populations. The structure of populations is represented by the tree in Figure 3a. We plot gene-expression of the lineage from the root to population 2. The number of EVFs is 20 for each of the three kinetic parameters, and 12 EVFs of parameter *s* are Diff-EVFs. σ is set to 0.4. (**e**) Heatmap of expression of genes with at least 4 Diff-EVFs along the lineage from the root to population 2.

Supplementary Figure 4

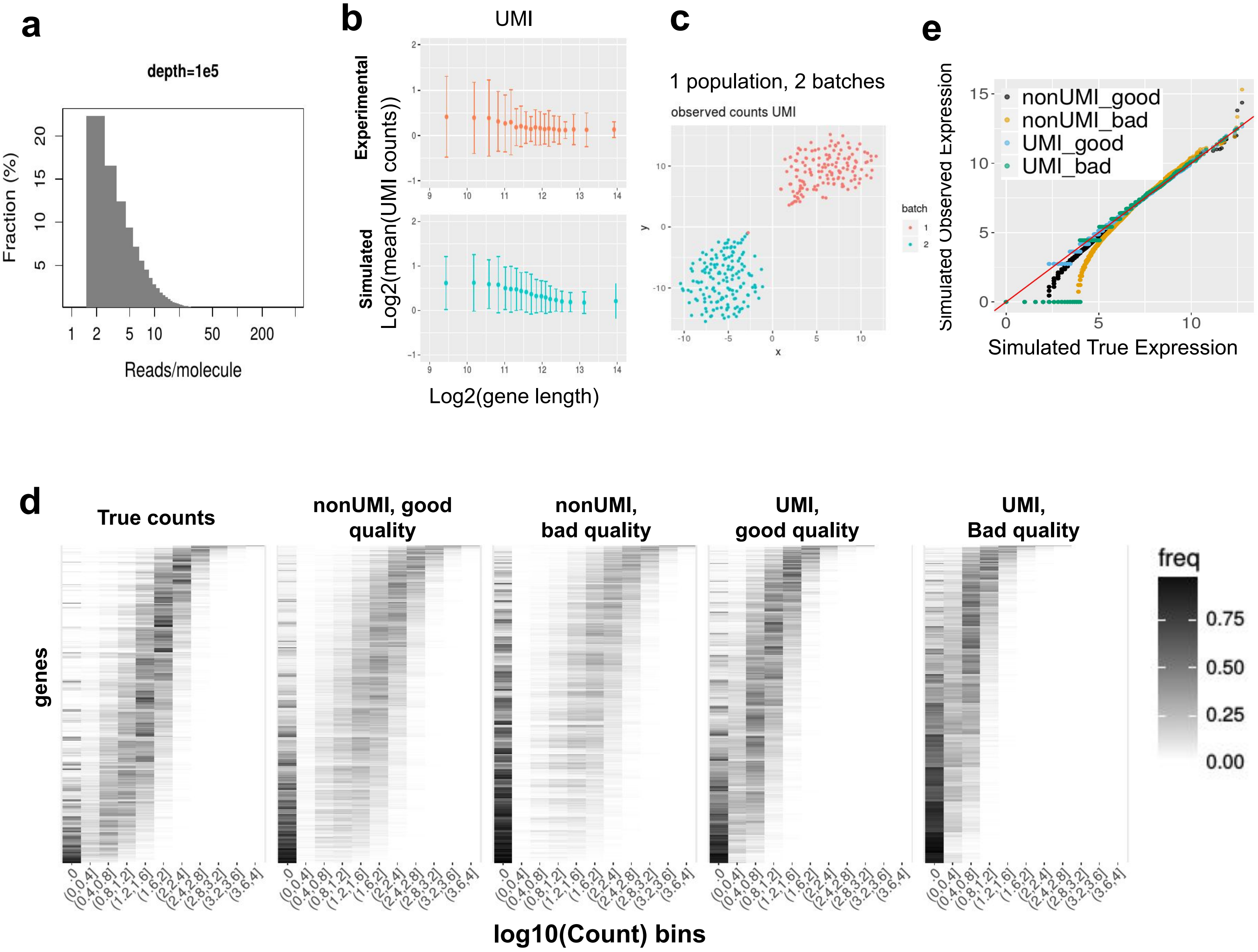

Supplementary Figure 4 Simulation of technical variation. (**a**) The distribution of number of reads per UMI sequenced in our simulated data, when using the UMI protocol, with 100k reads per cell. (**b**) The gene length bias in observed counts with UMI protocol, respectively from experimental and simulated data. The experimental data is the cortex dataset from paper: Zeisel, A. *et al. Science* **347**, 1138–1142 (2015). (**c**) TSNE plot of cells simulated for one homogeneous population in two batches. (**d**) The histogram heatmap of gene expression of true simulated counts, and observed simulated counts under "good" and "bad" parameter settings. The parameters are the same as described in Figure 4c. In these heatmaps, each row corresponds to a gene, each column corresponds to a level of expression, and the color intensity is proportional to the number of cells that express the respective gene at the respective expression level. (**e**) Q-Q plots of gene expression of true simulated counts and observed simulated counts under "good" and "bad" parameter settings. The data is the same as that plotted in Figure 4c.

Supplementary Figure 5

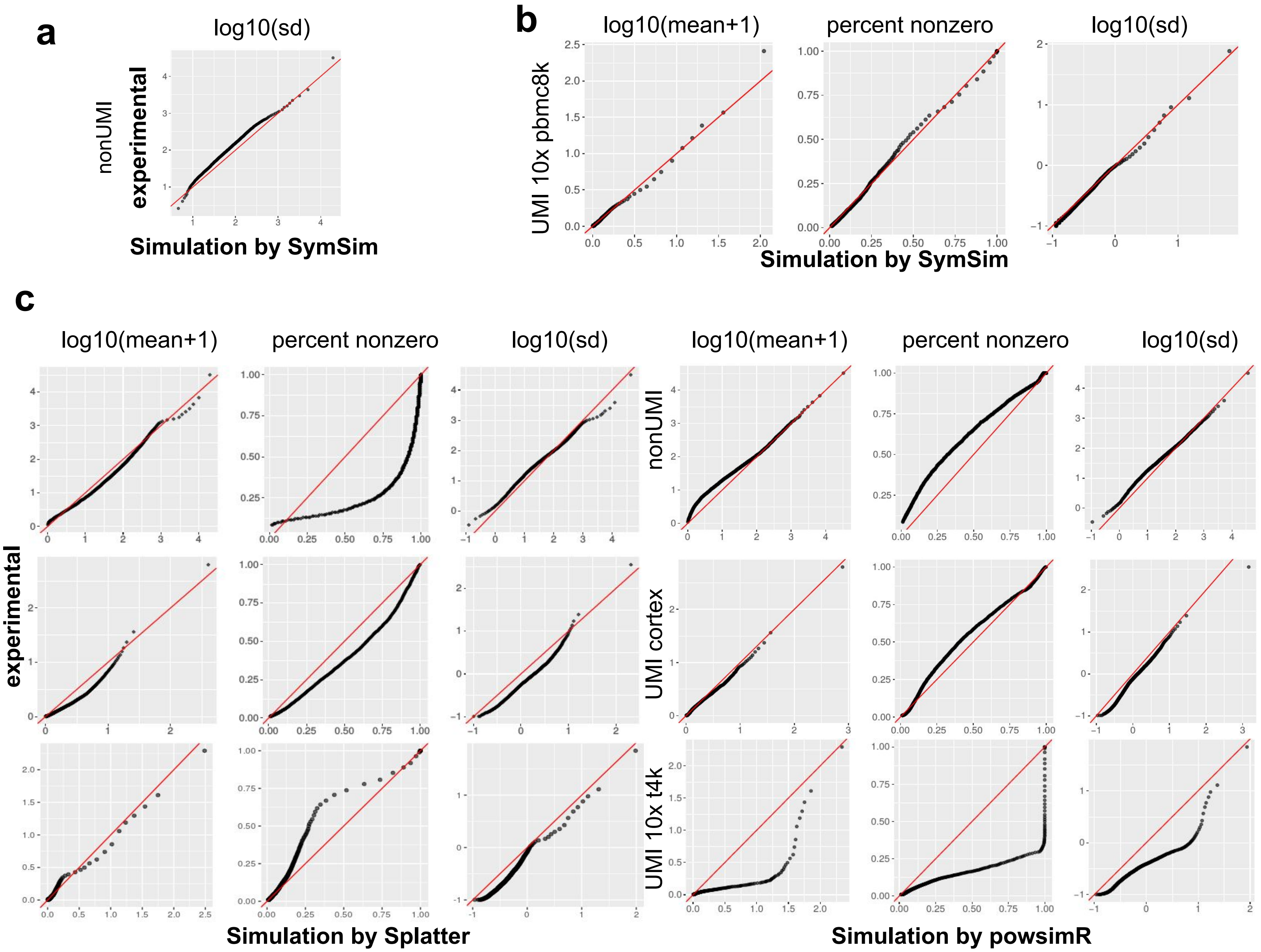

Supplementary Figure 5

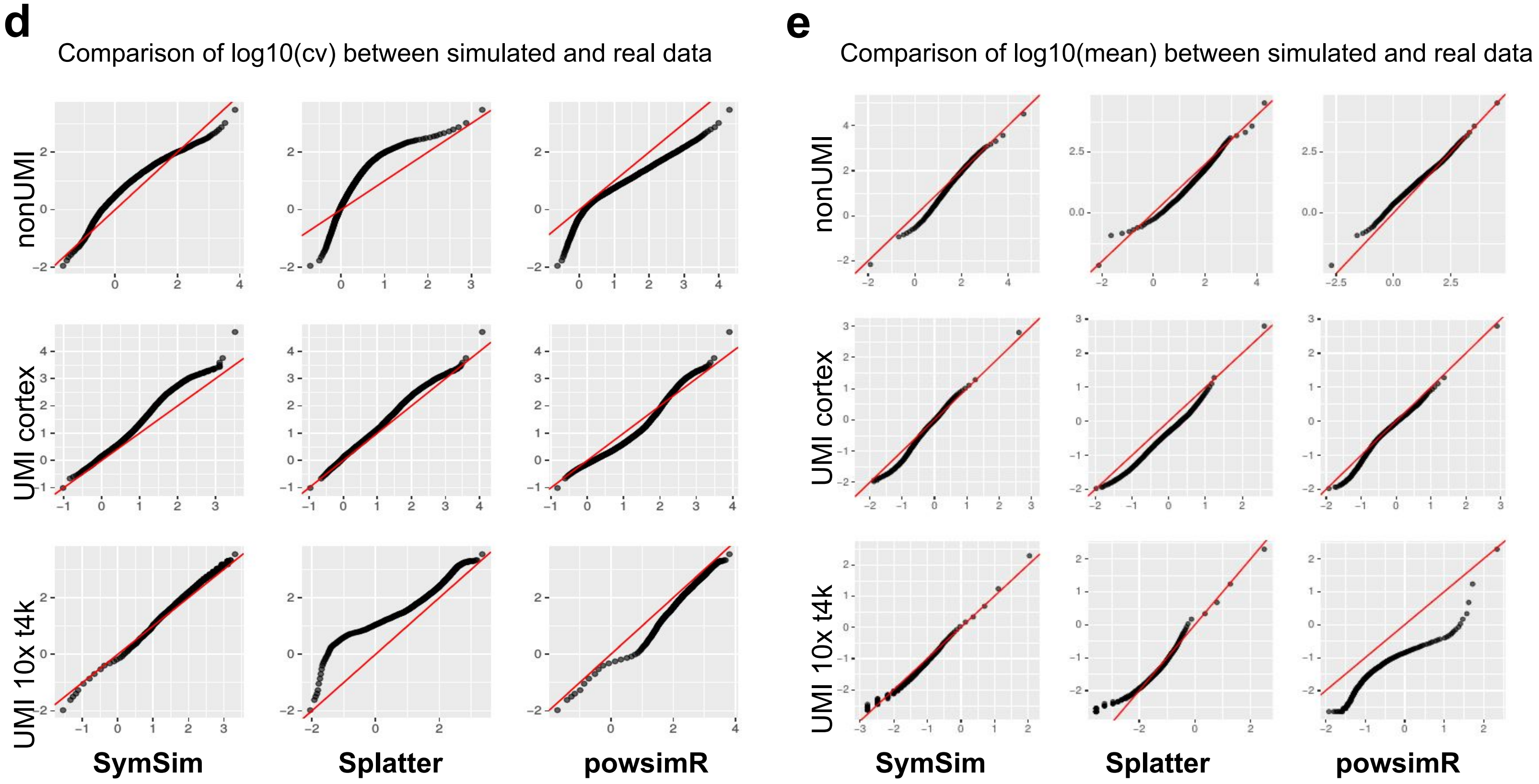

Supplementary Figure 5 Comparison of SymSim and existing simulators with various measurements. (**a**) Q-Q plots of standard deviation for genes between experimental data and SymSim simulated data for the non-UMI protocols after removing a proportion of lowly expressed genes. (**b**) Q-Q plots of mean, percent-nonzero, and standard deviation between a second 10x dataset (pbmc8k) and SymSim simulated dataset. (**c**) Q-Q plots comparing the mean, percent non-zero and standard deviation in experimental counts and simulated observed counts respectively for the non-UMI, UMI cortex and UMI 10x t4k datasets. The simulation is performed by Splatter (left three columns) and powSimR (right three columns). (**d**) Q-Q plots comparing the coefficient of variation (CV) in experimental counts and simulated observed counts respectively for the non-UMI, UMI cortex and UMI 10x t4k datasets. Simulation is performed by SymSim (left column), Splatter (middle column) and powSimR (right column). (e) Q-Q plots comparing the log10(mean) in experimental counts and simulated observed counts respectively for the non-UMI, UMI cortex and UMI 10x t4k datasets. Simulation is performed by SymSim (left column), Splatter (middle column) and powSimR (right column).

Supplementary Figure 6

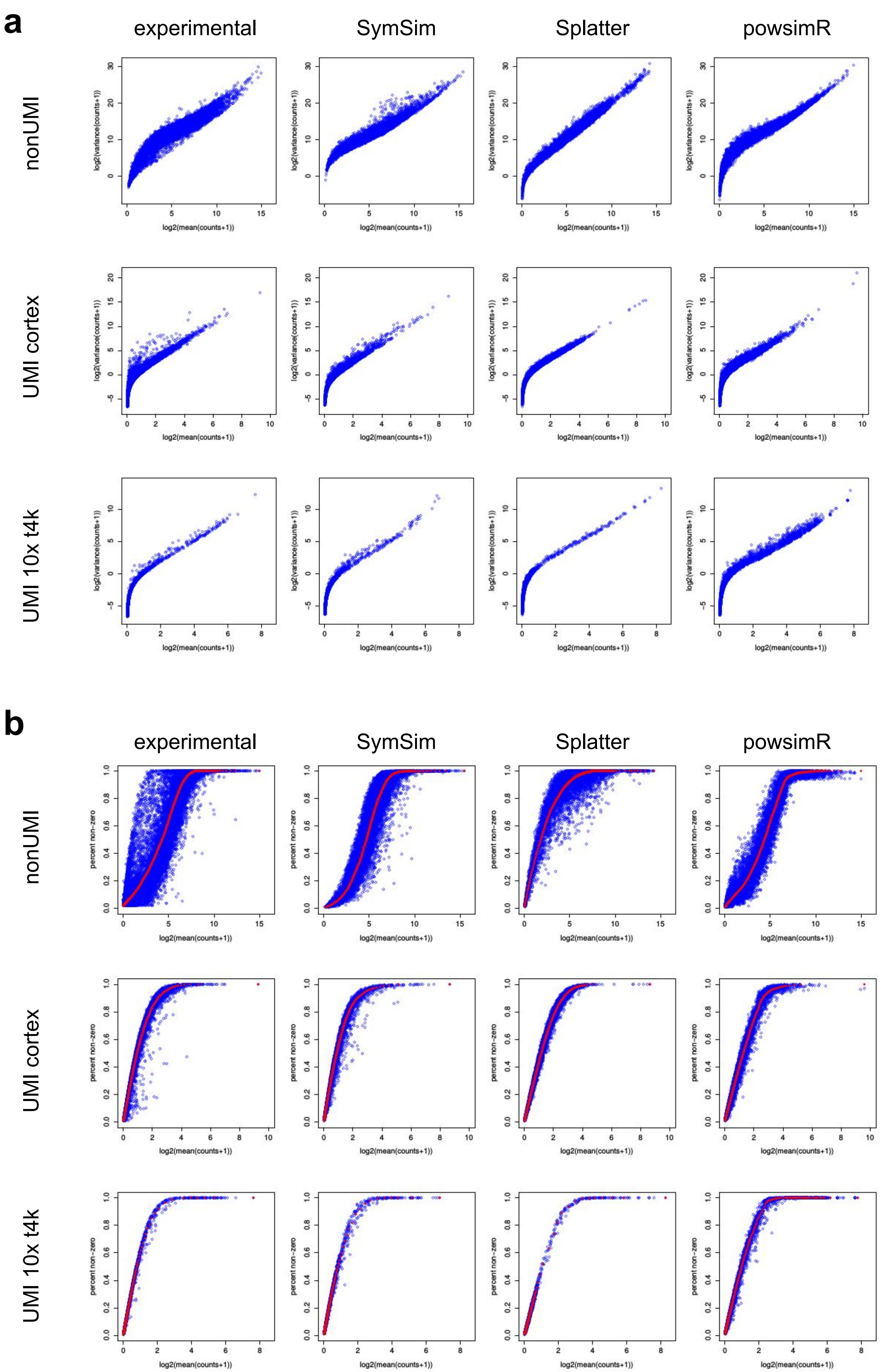

Supplementary Figure 6 Comparison of SymSim and existing simulators in terms of mean-variance relationship and mean *vs* percent-non-zero plots. (**a**) The mean-variance relationship of the three experimental datasets (non-UMI Th17 data, top row; UMI cortex data, middle row; UMI 10x t4k data, bottom row) and the corresponding simulated datasets by SymSim (2nd column), Splatter (3rd column) and powSimR (4th column). (**b**) The mean *vs* percent-non-zero plots of the three experimental datasets (non-UMI Th17 data, top row; UMI cortex data, middle row, UMI 10x t4k data, bottom row) and the corresponding simulated datasets by SymSim (2nd column), Splatter (3rd column) and powSimR (4th column). Red dots are the 0%, 0.2%, 0.4%, …, 100% percentiles of the mean and percent-non-zero values of all genes.

Supplementary Figure 7

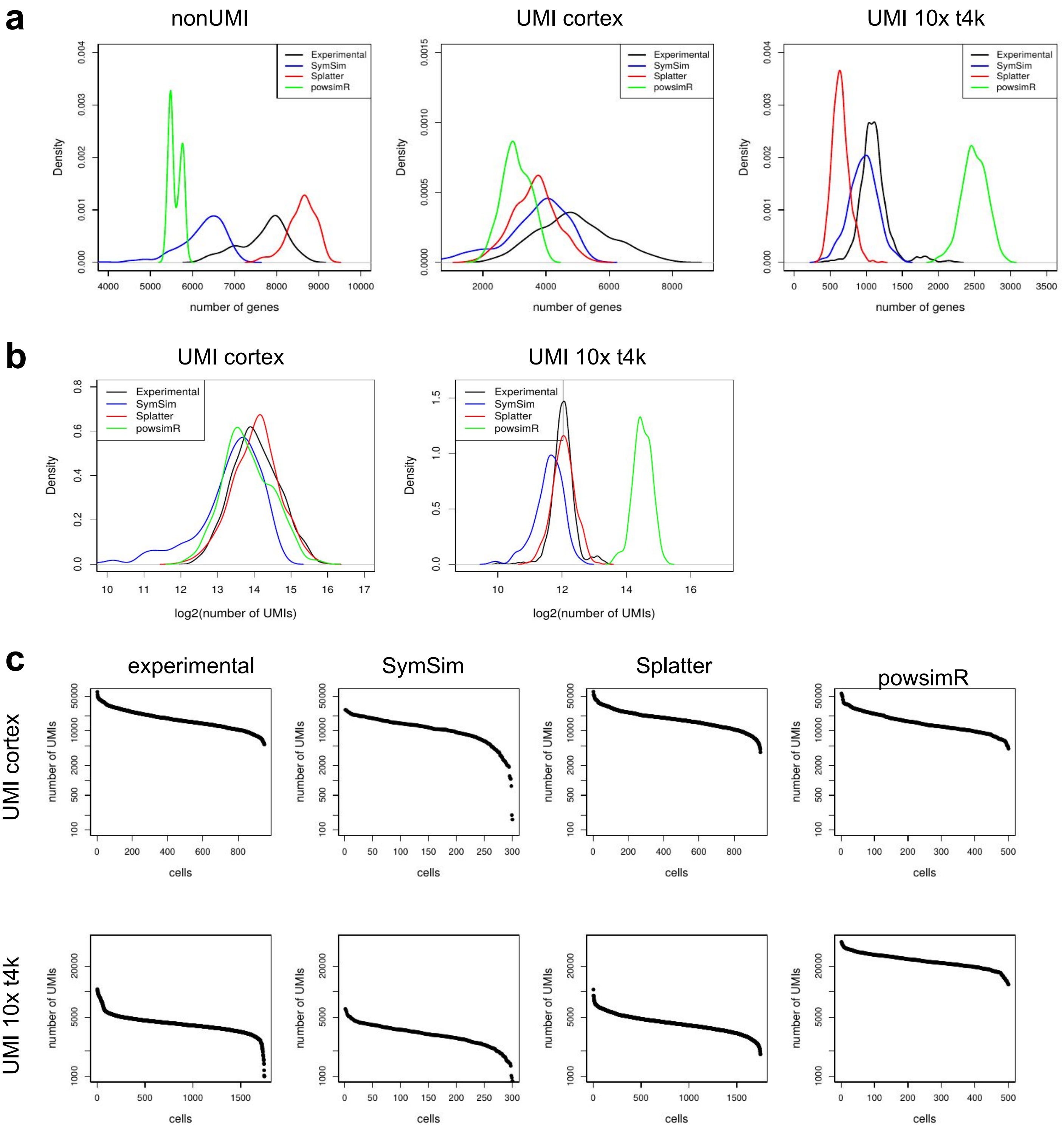

Supplementary Figure 7 Comparison of SymSim and existing simulators with various measurements. (**a**) Distribution of number of expressed genes per cell of the three experimental datasets (non-UMI Th17 data, left; UMI cortex data, middle; UMI 10x t4k data, right) and the corresponding simulated data by SymSim, Splatter and powSimR. (**b**) Distribution of number of UMIs per cell for the two UMI datasets and the corresponding simulated data by SymSim, Splatter and powSimR. (**c**) Number of UMIs per cell of the two UMI datasets and the corresponding simulated datasets, sorted in decreasing order.

Supplementary Figure 8

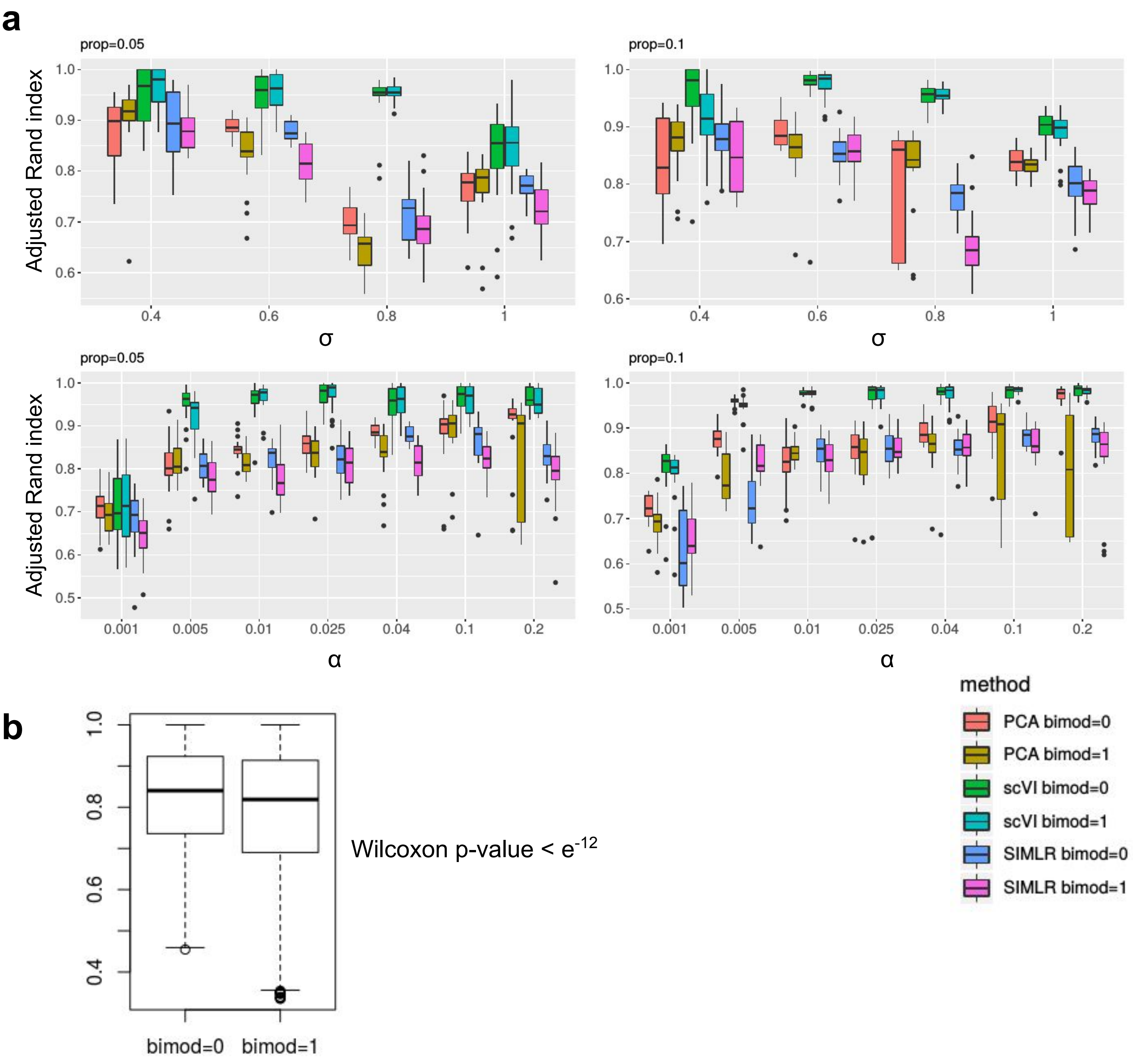

Supplementary Figure 8 Effects of *bimod* parameter on the performance of clustering methods. (**a**) Top row: ARI of the rare populations using the three clustering methods (PCA, scVI and SIMLR) with different values for *bimod* (*bimod*=0 and *bimod*=1) when changing σ (α=0.04). Left plot: the rare population accounts for 5% of all the cells; right plot: the rare population accounts for 10% of all the cells. Bottom row: ARI of the rare populations using the three clustering methods with different values for *bimod* when changing α (σ=0.6). Left plot: the rare population accounts for 5% of all the cells; right plot: the rare population accounts for 10% of all the cells. (**b**) Boxplots of aggregated ARI values, separated only by different values of *bimod*. Boxplots are plotted using the boxplot() function in R with default parameters, where the bottom and top of the box are the lower and upper quartiles respectively, and the band near the middle of the box is the median.

Supplementary Figure 9

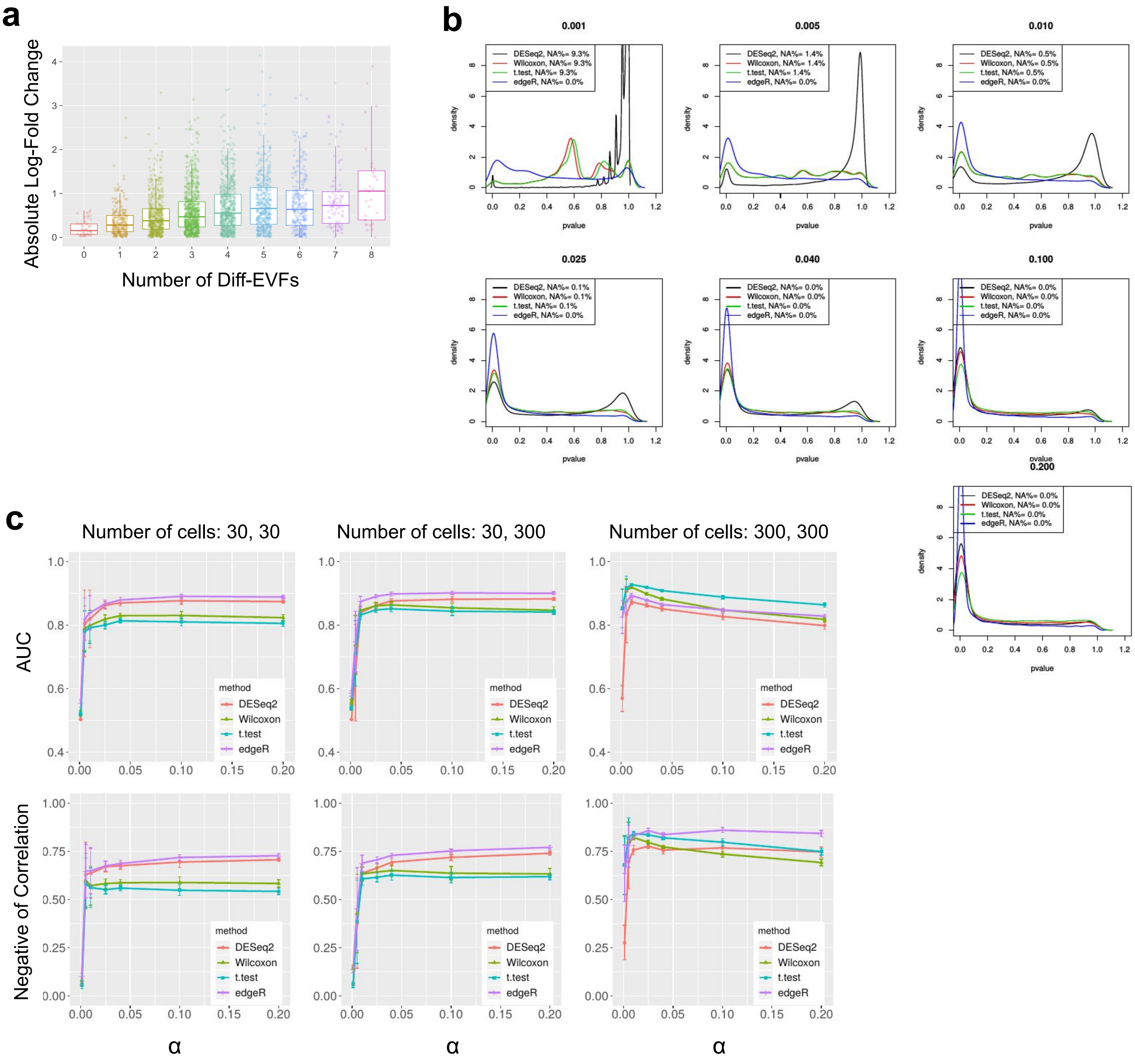

Supplementary Figure 9 Benchmarking differential expression methods. (**a**) The log fold change of genes with different number of Diff-EVFs. (**b**) The distribution of p-values of four different DE methods, with different values of capture efficiency α (noted as the title of each plot), with population size 300 vs 300. (**c**) The AUROC (top row) and negative of correlation (bottom row) measures of the four different methods for DE gene detection. In these plots the adjusted p-values from DESeq2 are used instead of the p-values, and the genes with NAs in the adjusted p-values are removed for all methods to calculate the accuracy measures. The numbers of cells in both populations for the plots on the left column are both 30, on the middle column the number of cells are respectively 30 and 300, and on the right column the numbers of cells are both 300.

Supplementary Figure 10

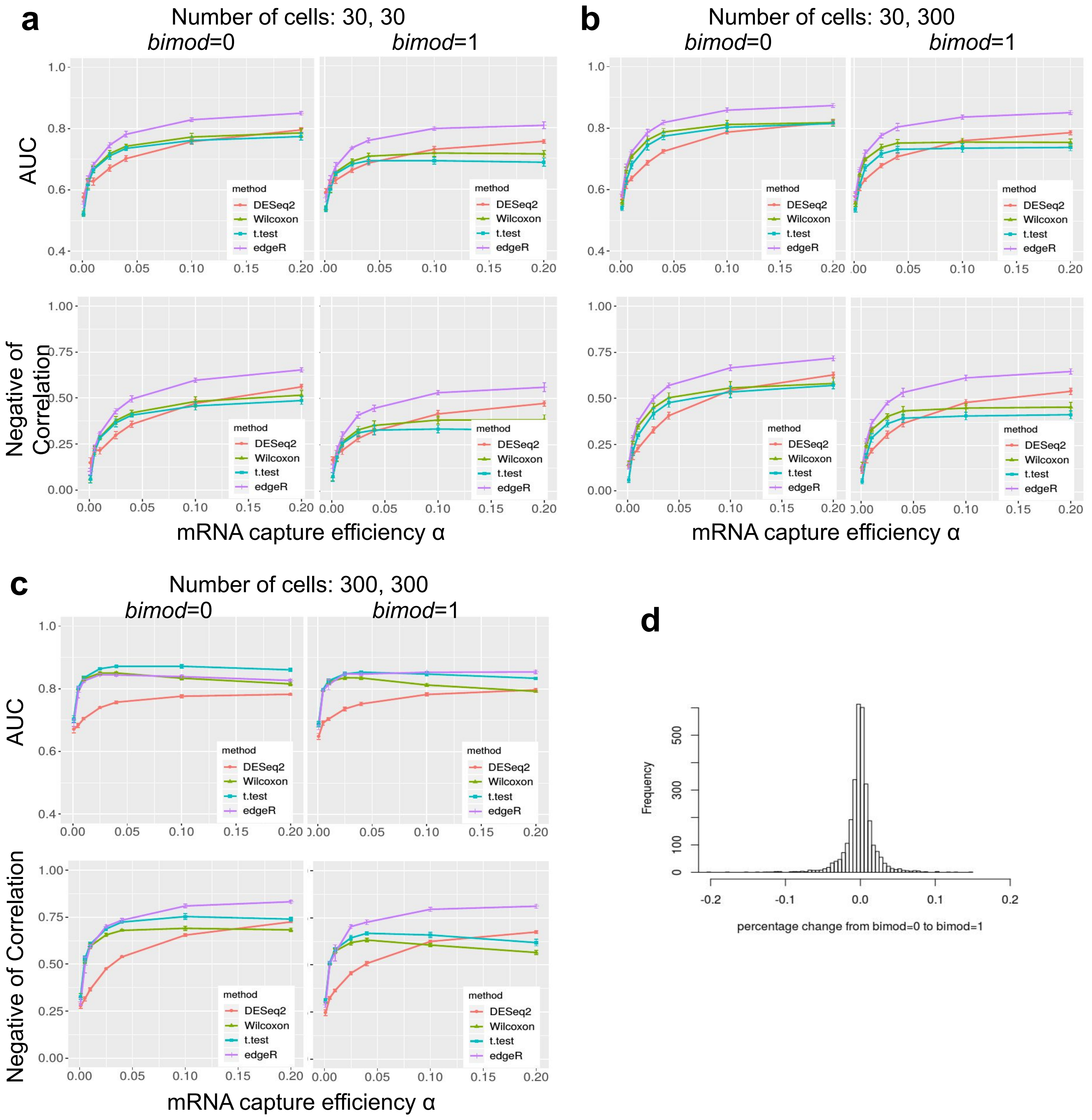

Supplementary Figure 10 Effects of *bimod* parameter on the performance of DE methods. In each panel, we show the AUROC (area under receiver operating characteristic curve, top row) and Negative of Correlation (bottom row) of detecting DE genes using four different methods from observed counts with changing capture efficiency *α* (σ=0.6). Left column corresponds to *bimod*=0 and right column corresponds to *bimod*=1. (**a**) Number of cells in the two populations (populations 2 and 4) are 30 and 30. (**b**) Number of cells in the two populations are 30 and 300. (**c**) Number of cells in the two populations are 300 and 300. (**d**) Percentage of change of total transcript for each gene from *bimod*=0 to *bimod*=1.

Supplementary Figure 11

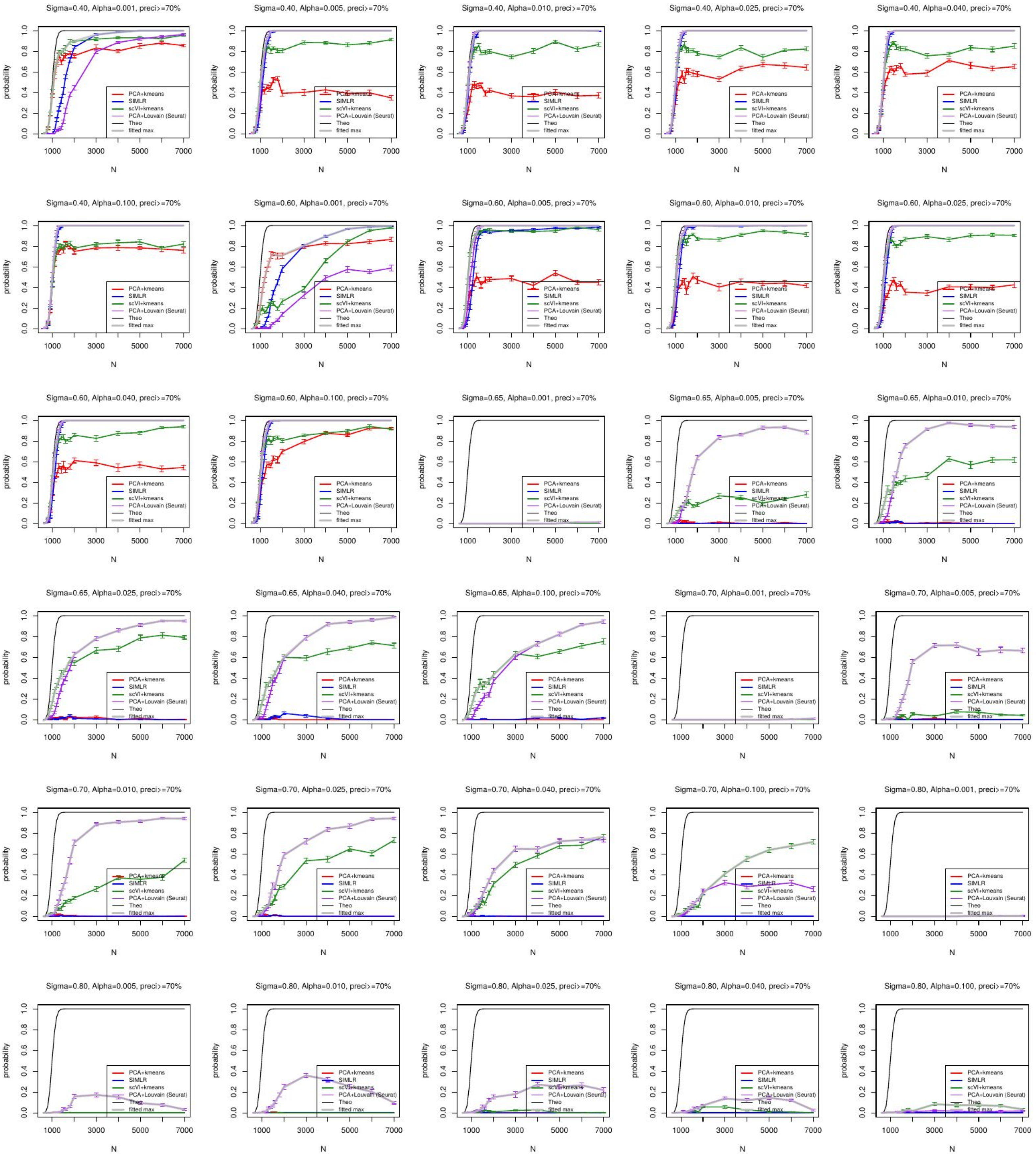

Supplementary Figure 11 The probability of detecting the rare population (population 2 in the tree shown in Figure 3) under a wide range of configurations of σ (Sigma) and α (Alpha). The criteria of detecting the population is that at least 50 cells are detected (true positive >=50) with precision at least 70%.

Supplementary Tables

Supplementary Table 1 Values or ranges of kinetic parameters from literature

Paper	Genes	Method	Parameters
Padovan-Merhar, O. and Raj, A. 2015. Single Mammalian Cells Compensate for Differences in Cellular Volume and DNA Copy Number through Independent Global Transcriptional Mechanisms. *Molecular Cell*. 58, 2 (2015), 339–352.	UBC, MYC, EEF2, TUSC3	smFISH<br><br>Transcriptional Blockage	Burst size 500 ~ 4000<br>log10(burst size): 2.7 ~ 3.6
Rabani, Michal, *et al*. "A Massively Parallel Reporter Assay of 3′ UTR Sequences Identifies In Vivo Rules for mRNA Degradation." *Molecular cell*, 2017	3' UTR sequences during early zebrafish embryogenesis	MPRA	half-life of mRNA is 1 ~ 10h<br>d: 0.0116 min-1 ~ 0.001 min-1
Bahar Halpern, K. *et al*. 2015 Bursty gene expression in the intact mammalian liver. *Molecular cell*. 58, 1 (Apr. 2015), 147–56.	Achy, Actb, Ass1, Fasn, G6pc, Pck1, Srebf1,Insr	smFISH	burst size 1-700<br>kon 0.01-1.32 hr^{-1}<br>koff 0.021-3.36 hr^{-1}<br>log10(burst size): 0 ~ 2.8
Skinner, SO *et al*. 2016. Single-cell analysis of transcription kinetics across the cell cycle. *Elife*. (2016).	Oct4,Nanog	smFISH	kon: 9×10^{-3} min^{-1} for Oct4<br>log10(kon/d): -0.04 ~ 0.9<br>kon: 2×10^{-3} min^{-1} for Nanog<br>log10(kon/d): -0.7 ~ 0.3<br>koff: 2×10^{-2} min^{-1} for Oct4<br>log10(koff/d): 0.3 ~ 1.3<br>7×10^{-3} min^{-1} for Nanog<br>log10(koff/d): -0.15 ~ 0.85
Dey, Siddharth S., *et al*. "Orthogonal control of expression mean and variance by epigenetic features at different genomic loci." *Molecular systems biology*, 11,5 (2015): 806.	HIV LTR	smFISH	S/k_{off} (Burst size): 2 ~ 24<br>log10(burst size): 0.3 ~ 1.38<br>k_{on}/d (Burst frequency): 0.3-4.5<br>log10(kon/d): -0.52 ~ 0.65
Singh, Abhyudai, *et al*. "Dynamics of protein noise can distinguish between alternate sources of gene-expression variability." *Molecular systems biology*, 8,1 (2012): 607.	HIV LTR	Transcriptional blockage, smFISH	S/k_{off} (Burst size): 2 ~ 12<br>log10(burst size): 0.3 ~ 1.1

Suter, D.M. *et al*. 2011. Mammalian genes are transcribed with widely different bursting kinetics. *Science*. 332, 6028, 472–4.<br><br>Value of d is taken from the average of range from Rabani *et al* 2017	Bmal1a, Glutaminase, Prl2C2, Serpine1, Sh3kbp1, Plectin1	smFISH	kon 0.02-0.06 min-1<br>log10(kon/d): 0.3 ~ 1.8<br>koff 0.1-0.6 min-1<br>log10(koff/d): 1.3 ~ 2<br>s 3-18 min-1<br>log10(s/d): 2.7 ~ 3.5<br>Burst size: 1 ~ 35<br>log10(Burst size): 0 ~ 1.54
Christoph Fritzsch *et al*. Estrogen-dependent control and cell-to-cell variability of transcriptional bursting. *Molecular systems biology.* (2018), 14, 2: e7678--e7678	GREB1	Live cell imaging	Burst size: 1 ~ 50<br>log10(Burst size): 0 ~ 1.7
Hendy, Oliver, *et al*. "Differential context-specific impact of individual core promoter elements on transcriptional dynamics." *Molecular biology of the cell* 28.23 (2017): 3360-3370.	MHC class 1 genes	smFISH	S/k_{off} (Burst size): 5.5 ~ 13.9<br>log10(burst size): 0.74 ~ 1.14<br>k_{on}/d: 4.4-13.9<br>log10(kon/d): 0.64 ~ 1.14
Summary from all above literature			log10(kon): -0.7 ~ 1.8<br>log10(koff): -0.15 ~ 2<br>log10(s): 2.7 ~ 3.5<br>log10(burst size): 0 ~ 3.6

Supplementary Table 2 Top parameters which match the nonUMI Th17 dataset

Gene_effects_sd	Sigma (σ)	scale_s	Alpha_mean (α)	Alpha_sd (β)	Depth_mean (*Depth*)	Depth_sd (*Depth_sd*)	nPCR1	prop_hge	mean_hge
1	0.2	0.5	0.15	0.045	2e6	6e5	14	0.025	4
1	0.2	0.5	0.15	0.045	2e6	6e5	18	0.02	4
2	0.1	0.3	0.2	0.06	2e6	6e5	18	0.02	4
2	0.1	0.3	0.2	0.06	2e6	6e5	18	0.025	4
1	0.2	0.5	0.15	0.045	2e6	6e5	18	0.025	4

Supplementary Table 3 Top parameters which match the UMI Cortex dataset

Gene_effects_sd	Sigma (σ)	scale_s	Alpha_mean (α)	Alpha_sd (β)	Depth_mean (*Depth*)	Depth_sd (*Depth_sd*)	nPCR1	prop_hge	mean_hge
2	0.2	0.4	0.05	0.025	3e5	1.5e4	14	0.01	3
2	0.2	0.4	0.05	0.025	3e5	1.5e4	14	0.02	3
2	0.2	0.5	0.03	0.009	5e5	1.5e4	10	0.03	4
2	0.2	0.5	0.03	0.009	3e5	9e4	10	0.02	4
2	0.2	0.4	0.05	0.015	3e5	9e4	14	0.02	3

Supplementary Table 4 Top parameters which match the UMI 10x t4k dataset

Gene_effects_sd	Sigma (σ)	scale_s	Alpha_mean (α)	Alpha_sd (β)	Depth_mean (*Depth*)	Depth_sd (*Depth_sd*)	nPCR1	prop_hge	mean_hge
2	0.2	0.3	0.007	0.0021	95000	28500	14	0.015	6
2	0.2	0.3	0.007	0.0007	70000	7000	14	0.015	6
2	0.2	0.1	0.02	0.004	70000	14000	14	0.015	6
2	0.2	0.1	0.02	0.006	70000	21000	10	0.015	6
2	0.2	0.2	0.01	0.003	70000	21000	14	0.015	6

Supplementary Table 5 Top parameters which match the UMI 10x pbmc8k dataset

Gene_effects_sd	Sigma (σ)	scale_s	Alpha_mean (α)	Alpha_sd (β)	Depth_mean (*Depth*)	Depth_sd (*Depth_sd*)	nPCR1	prop_hge	mean_hge
2	0.2	0.1	0.02	0.004	95000	19000	10	0.015	6
2	0.2	0.3	0.007	0.0021	95000	28500	14	0.015	6
2	0.2	0.1	0.02	0.006	95000	28500	10	0.015	6

2	0.2	0.3	0.007	0.0014	95000	19000	10	0.015	6
2	0.2	0.1	0.02	0.004	95000	19000	14	0.015	6

Supplementary Table 6 Parameters used for regression in Figure 5a

Parameter	Values	# simulations per value
N (total number of cells)	1000 2000 4000 6000 8000	1440
Prop (proportion of rare population)	0.01 0.03 0.05 0.10 0.20	1440
σ (within population variability)	0.4 0.6 0.8 1.0	1800
α (capture efficiency)	0.001 0.005 0.010 0.025 0.050 0.100	1200
Depth (sequencing depth)	5e+03 1e+04 5e+04 1e+05	1800
standard deviation (SD) of α	5e-04	7200
standard deviation (SD) of Depth	1000	7200

Supplementary Table 7 Parameters used for benchmarking of DE in Figure 6 and for trajectory inference methods analysis in Figure 7

Parameter	Values
nevf	30
n_de_evf (# Diff-EVFs for *s* only)	18
σ (within population variability)	0.2 0.4 0.6 0.8 1.0
α (capture efficiency)	0.001 0.005 0.010 0.025 0.05 0.1 0.2
Depth (sequencing depth)	1e+05

Supplementary Table 8 Parameters used for “Experimental Design” analysis in Figure 8

Parameter	Values
Prop (proportion of rare population)	0.05
n_de_evf (# Diff-EVFs for *s* only)	18
σ (within population variability)	0.2 0.4 0.6 0.65 0.7 0.8
α (capture efficiency)	0.001 0.005 0.010 0.025 0.05 0.1
Depth (sequencing depth)	1e+05

Supplementary Notes

Supplementary Note 1: Kinetic parameters from experiments

The goal of performing kinetic parameters estimation from real data is obtain realistic ranges of plausible parameter values. The ranges in the distributions we obtain (Figure 2b) are in line with observations from other experiments using smFISH or transcription inhibition based methods to measure kinetic parameters (Supplementary Table 1). When we plot the distributions of log10(burst size) and log10(burst frequency) (Supplementary Fig. 3a), the ranges we get are similar to those reported by Larsson *et al* (Larsson et al. “Genomic Encoding of Transcriptional Burst Kinetics.” *Nature* 565 (7738): 251–54. 2019). In their Figure 1c, the range of log10(burst frequency) is (-2, 1), which is where our main mode falls. In their Figure 1d, the range of log10(burst size) is (0, 2.5), which is close to our (-0.2, 3) (Supplementary Fig. 3a). Through simulations we show that with our procedure, we can gain comparable accuracy in estimating the distributions of parameters using imputed counts as using true counts, and both imputed and true counts yield similar ranges of the parameters to the true parameters (Supplementary Fig. 3b, Methods).

Supplementary Note 2: Setting branch lengths and σ to get clusters of different clusterability

In the function of generating multiple discrete populations, users can control the extent of between-population variation by setting the branch lengths of the input tree, and control the within-population variation by parameter σ. Notably, both σ and the square root of branch lengths in the tree are in units of EVF values. For any given Diff-EVF and any two given populations, the ratio of square root tree distance to σ determines the overlap between the distributions of the two Diff-EVFs. Thus this ratio determines the separability between the two populations. Take the Diff-EVF1 of populations 2 and 3 in Figure 3 as an example: we can show that

$$E(|y_2(1) - y_3(1)|) = \sqrt{d_{23}} \cdot \sqrt{2/\pi} \qquad (1)$$

where d_{23} is the distance in the tree between Populations 2 and 3. As the EVF values of Diff-EVF1 for cells in Populations 2 and 3 are sampled respectively from distributions $N(y_1(1),\sigma^2)$

and $N(y_1(2),\sigma^2)$ (Figure 3), the ratio $H = \frac{E(|y_1(1)-y_2(1)|)}{\sigma} = \frac{\sqrt{d_{23}}\cdot\sqrt{2/\pi}}{\sigma}$ correlates with the separability between cells from Population 2 and cells from Population 3. Detailed derivation and proof of Supplementary Equation 1 are in Supplementary Note 7 at the end of this document.

Supplementary Note 3: Distributions of number of fragments

When simulating the fragmentation step, we need the number of fragments obtained from a transcript. This number is dependent on the transcript length (denoted by *L*), the read length (*r*), maximum fragment length (*f*) and expected gap size (*g*) of the reads assuming we use paired-end sequencing. The fragmentation efficiency which is the probability with which a cut happens to a position on the transcript is: *e=1/(2*r+g)*.

For nonUMI protocols where full length mRNA is sequenced, for each transcript length, we simulate the fragmentation process many times with the probability *e* and remove resulting pieces which have length smaller than *r* or greater than *f*, and we obtain a distribution of number of valid fragments for a given transcript length. In SymSim, we just sample from this distribution.

For UMI protocols, we only need the valid fragments at the 3' end. In this case, we can derive theoretical distributions of the probability that a mRNA copy gives rise to a fragment. The expressions are as follows:

$$\begin{cases} (1-e)^r(1-(1-e)^{(f-r-1)}), & \text{if } L \geq f \\ (1-e)^r, & \text{if } r < L < f \\ 0, & \text{if } L \leq r \end{cases} \quad (2)$$

So during SymSim we sample with these probabilities to get the number of 3' end fragments (which will be either 0 or 1).

In our paper, we set *r*=100, *f*=1000, *g*=200.

Key parameters which give rise to the length bias patterns shown in Figure 4b are: α=0.05, *lenslope*=0.023, nbins=20, *MaxAmpBias*=0.3, *Depth*=1.3e6.

Supplementary Note 4: Top parameters which give rise to simulated datasets similar to real data sets

With SymSim we generate a database where datasets are simulated with a large grid of parameters. We also calculate a "summary" for the dataset corresponding to each parameter configuration. This summary includes mean expression, percentage of expressing cells, and

Fano factor for each gene. Given an experimental dataset, one can use these statistics to find the best matching simulations in our database. The parameters which yield the best matching simulations can give us insights on the properties of the experimental dataset. In Supplementary Tables 2-5 we show the top 5 parameter configurations which match best to respectively: the nonUMI Th17 dataset, the UMI Cortex dataset (subpopulation CA1 pyramidal neuron cells), the UMI 10x t4k dataset (subpopulation1), the UMI 10x pbmc8k dataset (subpopulation1). We see that in general the Th17 dataset has highest capture efficiency (Alpha_mean), followed by the UMI Cortex dataset, and the 10x datasets have lowest capture efficiency.

The parameters we keep fixed for all UMI and non-UMI datasets are: *nevf*=30, *evf_center*=1, *geffect_mean*=0, *gene_effect_prob*=0.3, *bimod*=0, *lenslope*=0.023, *nbins*=20, *MaxAmpBias*=0.3, *rate_2PCR*=0.7, *nPCR2*=10, *nbatch*=1.

Supplementary Note 5: Additional comparison of simulators in generating datasets that resemble read data

As additional comparisons of SymSim, Splatter and powsimR, we plot the mean *vs* variance relationship (Supplementary Fig. 6a) and mean *vs* percent-nonzero relationship (Supplementary Fig. 6b) of genes for the experimental datasets and simulated datasets with each simulator. For both types of plots, the datasets simulated by SymSim are the most similar to the corresponding experimental datasets. Finally, we inspected the distributions of number of expressed genes per cell (Supplementary Fig. 7a), distributions of number of UMIs per cell (Supplementary Fig. 7b) and number of UMIs per cell sorted in decreasing order (Supplementary Fig. 7c). SymSim and Splatter are comparable in terms of these measures and fit experimental data better than powSimR. The difference between the distributions of powsimR and those of real data in Figures S7a-b can be due to that powsimR does not attempt to reproduce unfiltered count matrices, but instead outputs data after filtering of genes and cells. Notably, we limit our parameters to the values that are available in our pre-computed grid, which explains the possible differences between observed quantities (e.g., sequencing depth per cell) in SymSim and in the real-data.

Supplementary Note 6: Effects of bimodality on clustering and differential expression performance

To investigate the effect of bimodality on the performance of clustering algorithms, we increased the parameter *bimod* from 0 to 1 for the same datasets used in the analysis of Section "Using SymSim to evaluate clustering methods" and performed clustering. The comparison of clustering results between different methods and different values of *bimod* is shown in Supplementary Fig. 8a. We see that in most cases there is a decrease of performance for the same method with increase of *bimod*. We then aggregate all the values of adjusted Rand index for all methods and all the parameters of α and σ, but only group them by the *bimod* value, and performed Wilcoxon test between the two groups of values. Supplementary Fig. 8b shows that the difference between these two groups is small but significant (p-value < e^{-12}).

We then perform all DE methods on the datasets with *bimod*=1 and compare the results with the original dataset with *bimod*=0, used in Section "Using SymSim to evaluate differential expression methods" (Supplementary Fig. 10a-c). From Supplementary Fig. 10a-c we can see a drop in performance when increasing *bimod*, especially when the number of cells is small (Supplementary Fig. 10a-b). The drop is less prominent when the number of cells in the two populations are respectively 300 and 300 (Supplementary Fig. 10c). Notably, the drop in the performance of clustering and DE performance cannot be simply attributed to a global decrease in gene expression levels, since increasing *bimod* does not change this statistic (Supplementary Fig. 10d and Methods).

Supplementary Note 7: Proof on relationships between branch lengths and within-population variable σ

1. Diagram of the tree
   R: EVF value at the root of the tree, for simplicity R=0
   W, X, Y: EVF value at the tips of the tree
   Z: EVF value at the most recent common ancestor of two populations.
   a, b, c, d: Branch length
   Let EVF values perform Brownian Motion with constant rate 1 for time equal to the branch length along the tree. The random variables at the tip of the tree represent the EVF population mean.

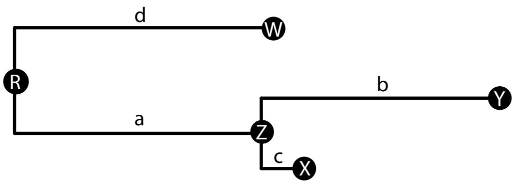

2. Relationship between Branch Length and EVF-Mean Variance-Covariance Matrix

   (a) EVF values at the tips of the tree are normally distributed with mean μ and variance equal to the sum of branch lengths between the tips and the root, with μ being the value at the root.
   Without loss of generosity, let $\mu = 0$

   $$W \sim N(0, d)$$

   We can reformulate Brownian Motion in terms of a 1 Dimensional Random Walk. In each unit time, EVF values take k steps of size $\frac{1}{\sqrt{k}}$ in random directions. Let the direction of the step be δ and it can take either value 1 or -1.

   $$W = \frac{1}{\sqrt{k}} \sum_{i=1}^{dk} \delta_i$$

   This can be re-written as

   $$W = \sqrt{d}(\frac{1}{\sqrt{dk}} \sum_{i=1}^{dk} \delta_i) \tag{3}$$

   Because δ_i are i.i.d with mean 0 and variance 1, by central limit theorem their normalized sum $\frac{1}{\sqrt{dk}} \sum_{i=1}^{dk} \delta_i$ has standard normal distribution. Thus

   $$W \sim N(0, d)$$

   When $\mu \neq 0$, $W = \mu + \frac{1}{\sqrt{k}} \sum_{i=1}^{dk} \delta_i$, so $W \sim N(\mu, d)$

(b) Covariance of the EVF values at the tips of the tree are equal to the branch length between the root and their most recent common ancestor.

$$Cov(X, Y) = E[(X - E[X])(Y - E[Y])]$$
$$= E[XY - XE[Y] - YE[X] + E[X]E[Y]] \tag{4}$$

Given the last result, we know that $E[X] = E[Y] = 0$

$$Cov(X, Y) = E[XY]$$

$$E[XY] = E[(Z + (Y - Z))(Z + (X - Z))]$$
$$= E[Z^2 + Z(X - Z) + Z(Y - Z) + (X - Z)(Y - Z)]$$
$$= E[Z^2] + E[Z(X - Z)] + E[Z(Y - Z)] + E[(X - Z)(Y - Z)] \tag{5}$$

(c) Claim: Expectation of the product of two independent normal variable with expectation 0 is also zero.
Proof: Let X and Y be two independent normal variable with mean 0 and standard deviation σ_x, σ_y. The expectation of their product can be written as

$$E[XY] = \int_{-\infty}^{\infty}\int_{-\infty}^{\infty} XYf(X, Y)dXdY \tag{6}$$

Because of independence,

$$E[XY] = \int_{-\infty}^{\infty}\int_{-\infty}^{\infty} XYf(X)f(Y)dXdY$$

By moving all terms independent of X outside of the inner integral,

$$= \int_{-\infty}^{\infty} Yf(Y)\int_{-\infty}^{\infty} Xf(X)dXdY$$

We know that the expection of X is equal to 0, so

$$\int_{-\infty}^{\infty} Xf(X)dX = 0$$

$$\int_{-\infty}^{\infty} Yf(Y) * 0 \; dY = 0$$

Thus $E[XY] = 0$. Due to the memoriless property of random walk, $Z, (X-Z)$, $Z, (Y-Z)$ and $(Y - Z), (X - Z)$ are all independent. By the property shown in part a

$$E[Z] = E[X - Z] = E[X - Z] = 0$$

thus

$$E[XY] = E[Z^2] = E[Z^2] - E[X]^2 = Var[Z] = a \tag{7}$$

3. Average Distance between EVF-Mean in Two Populations Separated
The average distance, or absolute differences between two populations can be expressed as the $E[|X - Y|]$. It can then be written as

$$E[|(X - Z) + (Z - Y)|]$$

From proof 1a, we know that $X|Z \sim N(Z, c)$,$Y|Z \sim N(Z, b)$,so $(X - Z)|Z \sim N(0, c)$,$(Z - Y)|Z \sim N(0, b)$. Because $X - Z|Z$ and $Z - Y|Z$ are independent, the distribution of their difference given Z is $N(0, b + c)$
Claim: The sum between two independent normal variables X and Y is normally distributed with variance $\sigma_X^2 + \sigma_Y^2$. We can show this using characteristic functions.

$$\varphi_X(t) = \mathrm{E}\left(e^{itX}\right), \qquad \varphi_Y(t) = \mathrm{E}\left(e^{itY}\right)$$

By indepence we have

$$\varphi_{X+Y}(t) = \mathrm{E}\left(e^{it(X+Y)}\right)$$

$$\begin{aligned}\varphi_{X+Y}(t) = \varphi_X(t)\varphi_Y(t) &= \exp\left(it\mu_X + \frac{\sigma_X^2 i^2 t^2}{2}\right)\exp\left(it\mu_Y + \frac{\sigma_Y^2 i^2 t^2}{2}\right) \\ &= \exp\left(it(\mu_X + \mu_Y) + \frac{(\sigma_X^2 + \sigma_Y^2) i^2 t^2}{2}\right)\end{aligned} \tag{8}$$

Which is the characteristic function of a normal distribution with mean $\mu_X + \mu_Y$ and variance $\sigma_X^2 + \sigma_Y^2$. Since we care about the absolute value of their differences, we then need to calculate the absolute value of a normally distributed variable. For simplicity we write the integration for a normally distributed variable $R \sim N(0, \sigma^2)$

$$E[|R|] = \int_{-\infty}^{\infty} |R| f(R) dR$$

$$= \int_{-\infty}^{0} -R f(R) dR + \int_{0}^{\infty} R f(R) dR$$

The density function of a normally distributed variable X is

$$f(X) = \frac{1}{\sqrt{2\pi\sigma^2}} e^{-\frac{(X-\mu)^2}{2\sigma^2}}$$

$$E[|R|] = \int_{-\infty}^{0} -R \frac{1}{\sqrt{2\pi\sigma^2}} e^{-\frac{R^2}{2\sigma^2}} dR + \int_{0}^{\infty} R \frac{1}{\sqrt{2\pi\sigma^2}} e^{-\frac{R^2}{2\sigma^2}} dR \tag{9}$$

Define new variable $u = \frac{-R^2}{2\sigma^2}$. We then have

$$R = \sqrt{-2u\sigma^2}, \frac{dR}{du} = \frac{\sigma^2}{\sqrt{-2u\sigma^2}}$$

Thus

$$\int_{-\infty}^{0} -R \frac{1}{\sqrt{2\pi\sigma^2}} e^{-\frac{R^2}{2\sigma^2}} dR = \frac{1}{\sqrt{2\pi\sigma^2}} \int_{-\infty}^{0} -R e^{-\frac{R^2}{2\sigma^2}} du \frac{dR}{du}$$

$$= \frac{1}{\sqrt{2\pi\sigma^2}} \int_{-\infty}^{0} -\sqrt{-2u\sigma^2} e^u \frac{\sigma^2}{\sqrt{-2u\sigma^2}} du$$

$$= \sqrt{\frac{\sigma^2}{-2\pi}} \int_{-\infty}^{0} e^u = \sqrt{\frac{\sigma^2}{-2\pi}}$$

Similarly,

$$\int_0^{\infty} R \frac{1}{\sqrt{2\pi\sigma^2}} e^{-\frac{R^2}{2\sigma^2}} dR = -\sqrt{\frac{\sigma^2}{-2\pi}} \int_0^{\infty} e^u = \sqrt{\frac{\sigma^2}{-2\pi}} \tag{10}$$

Thus, $E[|R|] = \sqrt{\frac{2\sigma^2}{\pi}}$

Up to here, we have proven that

$$E[|X - Y||Z] = \sqrt{\frac{2(b+c)}{\pi}}$$

Because this expression do not depend on Z,

$$E[X - Y] = \int_{-\infty}^{\infty} f(Z) E[|X - Y||Z] dZ$$

$$= E[|X - Y||Z] \int_{-\infty}^{\infty} f(Z) dZ$$

$$= E[|X - Y||Z] = \sqrt{\frac{2(b+c)}{\pi}}$$

4. The probability of overlap of EVF distribution between two populations
   We use this result to calibrate the value of σ_{within} (within population variation) used in our simulation. The average distance of the per-cell EVF to the population mean EVF is equal to $\sqrt{\frac{2\sigma_{within}}{\pi}}$. In our simulation, σ_{within} is the same for each population. We can derive the amount of overlap as a function of the distance between the EVE mean and the amount of within population variation.
   For example, if $\sigma_{within} = \frac{a}{2}$, a being the distance between the population mean. We can solve for the point of intersetion of the two density functions

$$\frac{1}{\sqrt{2\pi\sigma^2}} e^{\frac{-x^2}{2\sigma^2}} = \frac{1}{\sqrt{2\pi\sigma^2}} e^{\frac{-(x-a)^2}{2\sigma^2}} \tag{11}$$

   When we solve for x, we see that the two density function intersects at $\frac{a}{2} = \sigma$ from the population means. For normal distributions, the probability of having values greater than $\mu + \sigma$ or smaller than $\mu - \sigma$ is 0.159. Thus the total amount of overlap between the EVF values of the two populations is 2*0.159=0.318. We can generalize this result to other values of σ because as long as the value of σ is equal for each population, the point of intersection is always the mean of the two population mean. The probability of overlap is then

$$p(X > \mu + \frac{a}{2}) + p(X \leq \mu - \frac{a}{2}) \tag{12}$$
